# Chinese College Students’ Stigmatization towards People with Mental Illness: Familiarity, Perceived Dangerousness, Fear, and Social Distance

**DOI:** 10.3390/healthcare12171715

**Published:** 2024-08-27

**Authors:** Xu-Hong Li, Yin-Ling Irene Wong, Qinglu Wu, Mao-Sheng Ran, Tian-Ming Zhang

**Affiliations:** 1 Department of Social and Behavioural Sciences, City University of Hong Kong, Hong Kong, China; anthan@link.cuhk.edu.hk; 2 School of Social Policy & Practice, University of Pennsylvania, Philadelphia, PA 16802, USA; ylwong@sp2.upenn.edu; 3 Institute of Advanced Studies in Humanities and Social Sciences, Beijing Normal University, Zhuhai 519087, China; qinglu-wu@hotmail.com; 4 Mental Health Center, West China Hospital, Sichuan University, Chengdu 610041, China; msrancd@outlook.com; 5 Department of Social Work, Shanghai University, Shanghai 200444, China

**Keywords:** college students, familiarity, fear, perceived dangerousness, social distance

## Abstract

Background: Attribution models have been examined in Western countries. However, little is known about the applicability of the attitude–emotion–behavior model within Chinese culture. This study aimed to examine the association between familiarity, perceived dangerousness, fear, and social distance towards persons with mental illness (PMI) in the Chinese context. Methods: An online cross-sectional survey was conducted from October to November 2022 in mainland China. A total of 1493 college students completed a questionnaire evaluating familiarity, perception of dangerousness, fear, and social distance regarding PMI. Path analysis was employed to validate the model proposed in this study. Results: Participants expressed moderate to high levels of stigma towards PMI. Familiarity was negatively associated with social distance (*p* < 0.01). Participants who perceived PMI as dangerous were more prone to exhibit a reaction of fear (*p* < 0.001), consequently leading to social distance (*p* < 0.01). However, the mediating effect of perceived dangerousness and fear on the relationship between familiarity and social distance was not significant (*p* > 0.05). Conclusions: The results of this study provide support for Corrigan’s attributional model of stigma in the Chinese context. Contact-based interventions for stigma reduction should emphasize multiple elements of contact, including the quality of contact, rather than familiarity.

## 1. Introduction

Severe and pervasive mental health problems were not only widely reported during the COVID-19 pandemic but have also prevailed in the post-pandemic era [1,2,3]. The stigma of mental illness, operationalized as prejudicial attitudes, negative emotional reactions, and discriminatory behaviors, is evident as a significant barrier to recovery [4,5]. Goffman defined stigma as “a deeply discrediting attribute” that reduces the stigmatized individual “from a whole and usual person to a tainted discounted one” (p. 3) [6]. Numerous studies have documented that stigma erodes the well-being of persons with mental illness (PMI) who have clinically significant impairments in cognition, emotional regulation, or behavior, such as a reduction in self-esteem, an increase in self-rejection and suicide ideation, a reluctance to seek professional treatment, and social isolation [7,8,9,10].

An attitude–emotion–behavior model rooted in Weiner’s attribution theory [11] suggested that an individual’s perception towards PMI would affect their emotional reaction and behavioral judgments towards them [12,13]. Associations between perceptions of dangerousness, fear, and social distance were documented in prior studies [14]. Furthermore, it was shown that those who have greater familiarity with PMI or have more knowledge and awareness of mental illness perceived less dangerousness and subsequently showed less fearful reactions [12,15,16]. Therefore, a pathway based on Corrigan’s attributional model of stigma, suggesting that familiarity affects social distance by two mediators—perception of dangerousness and fear—was extensively studied [17,18,19].

The stigma of mental illness is widespread in college students, including those in China [3,20]. Prior research pointed out that younger individuals were more inclined to express negative attitudes towards PMI than older individuals, despite the fact that some of them had more knowledge about mental illness [21,22]. A global survey across 65 countries found that medical students perceived PMI as dangerous despite their professional training [23]. Additionally, fear of dangerousness and violence was strongly related to low social acceptance in medical students [24]. Previous literature has identified significant associations between familiarity, perceptions of dangerousness, and social distance among college students [25,26]. The evidence among college students also supports the hypothesis that cultural attributions of mental illness might affect the stigmatizing reaction towards PMI [27].

Despite the pervasiveness of mental illness stigma across nations, the level of stigma in Eastern societies was found to be higher compared to Western countries [28]. However, most of the studies were conducted in Western countries, and the application of the attitude–emotion–behavior model in Eastern cultural contexts remain to be investigated. East Asian culture is deeply embedded in Confucian values, with the accentuation of collectivism, social harmony, family reputation, and the individual’s obligation to adhere to social and cultural norms [29,30,31]. A substantial body of literature shows that PMI in China have suffered from more salient and prevalent stigma in comparison to their counterparts in Western societies [29,30].

Expanding knowledge and insights into the stigma of mental illness among college students will contribute to opening new avenues for developing and implementing anti-stigma interventions, ultimately building more inclusive societies [31,32]. The purpose of this study is to test Corrigan and his colleagues’ model utilizing data from a sample of college students in Shanghai, China. It is hypothesized that (1) familiarity is negatively associated with perception of dangerousness, fear, and social distance; (2) perception of dangerousness is positively associated with social distance through fear; (3) fear is positively associated with social distance; and (4) perception of dangerousness and fear are mediators between familiarity and social distance.

## 2. Methods

### 2.1. Study Design, Participants, and Procedures

The data of the present study were obtained from a project named mental health stigma among college students in China. Chinese undergraduates and postgraduates aged 18 years or older from universities in Shanghai were recruited to participate in the survey from October 2022 to November 2022. A self-report questionnaire was created using an online platform (https://www.wjx.cn/, accessed on 28 July 2023), a commonly used online survey tool in China, and distributed through social media applications (e.g., Weibo, WeChat). IP address restriction technology was used to avoid duplication of responses such that each participant was allowed to complete the survey once. In terms of the mechanisms for motivation, each participant who completed the questionnaire would receive a reward of 20 CNY. Inclusion criteria for the participants in this study were as follows: (1) registered as a university or college student in Shanghai; (2) aged 18 years or above. Participants who reported a history of mental illness and who did not complete the questionnaire were excluded. The final sample of the present study consisted of 1493 participants. Participants provided informed consent before answering the survey questions. Ethical approval for the study was obtained from the Ethics Committee of Shanghai University (ECSHU: 2022-001).

### 2.2. Measurements

Familiarity with PMI was measured using the level-of-contact report (LCR) [33,34]. This scale contains 12 levels of intimacy in contact with PMI, ranging from the lowest level of contact (1 = ‘I have never observed a person that I was aware had a serious mental illness’) to the highest level of contact (12 = ‘I have a mental illness’). A higher score is indicative of a higher level of familiarity. This scale has been validated and studied in diverse populations, including the Chinese population [12,35,36]. The current study reported acceptable internal consistency (Cronbach’s alpha = 0.65) for the LCR.

Perception of dangerousness and fear were assessed separately by two items from the 9-item Attribution Questionnaire (AQ-9). Perception of dangerousness was measured by the question of “How dangerous would you feel the person is?”, and fear was assessed by the item of “How scared of the person would you feel?” [37]. Participants were required to rate each item on a 9-point Likert scale, ranging from 1 (strongly disagree) to 9 (strongly agree), after reading a vignette related to a person with mental illness. An acceptable level of internal consistency (Cronbach’s alpha = 0.67) for the AQ-9 was reported in a previous study [38].

Social distance was evaluated using the 7-item Social Distance Scale (SDS), which has been identified as a useful tool to assess intended discriminatory behaviors exhibited by respondents [34,39]. On each item, participants were asked to report their inclination to interact with a PMI in a variety of social situations on a 5-point scale scoring from 1 (definitely willing) to 5 (definitely unwilling), with a higher total score being suggestive of greater social distance (ranging from 7 to 35). The internal consistency of the SDS is satisfactory (Cronbach’s alpha: 0.86) [39]. In this study, the SDS achieved excellent internal consistency (Cronbach’s alpha = 0.92).

The sociodemographic characteristics of the participants were collected by a self-administered questionnaire, including gender (male, female), age (date of birth), educational attainment, hometown (urban area, rural area, others), and ethnicity (Han, minority).

### 2.3. Statistical Analysis

Data analysis was carried out using SPSS version 22 and Mplus 8.0. Descriptive analysis was utilized to illustrate the characteristics of participants. T-tests and ANOVA were employed to explore whether the main study variables differed according to participants’ sociodemographic characteristics. Pearson correlation analysis was conducted to identify the bivariate association between the main study variables (familiarity, perception of dangerousness, fear, and social distance). Structural equation modeling (SEM) was employed to test the proposed path model concerning social distance. Sociodemographic variables were controlled when the model was examined. To assess the goodness-of-fit of the model, several indices were calculated [40]: root mean square error of approximation (RMSEA < 0.08), comparative fit index (CFI > 0.9), and Tucker–Lewis index (TLI > 0.9).

## 3. Results

### 3.1. Characteristics of Participants

A total of 1493 college students were recruited, of which 44.5% were males and 55.5% were females. The age of the students ranged from 18 to 40, with M = 21.53 (SD = 2.3). Overall, 31.3% were postgraduate students and 65.6% reported that their hometowns were in urban areas. The vast majority (96.6%) were of Han ethnicity. As shown in Table 1, the college students in the sample had low familiarity with PMI. The scores for perception of dangerousness and fear were both above the neutral score of 4.5 (scored 1–9), indicating moderate to high levels of perceived dangerousness and fear towards PMI [41,42]. Furthermore, they displayed moderate to severe (mean = 22.51) social distance towards PMI.

### 3.2. Differences in Main Variables among Students

As shown in Table 2, older students perceived more dangerousness (*p* < 0.05) and displayed more fear (*p* < 0.05) towards PMI. Male students markedly displayed greater familiarity (*p* < 0.05) and less social distance (*p* < 0.001) towards PMI. Moreover, postgraduate students displayed more fear (*p* < 0.01) and greater social distance (*p* < 0.001) towards PMI, while they displayed lower levels of familiarity (*p* < 0.001).

### 3.3. Bivariate Associations among Variables Hypothesized in the Model

Table 3 shows the results of the bivariate correlation analysis on the main variables (familiarity, perception of dangerousness, fear, and social distance) included in the proposed path model. Social distance had significant positive correlations with perception of dangerousness (*r* = 0.065, *p* < 0.05) and fear (*r* = 0.083, *p* < 0.01). Familiarity was negatively associated with social distance (*r* = −0.194, *p* < 0.01) and perception of dangerousness (*r* = 0.063, *p* < 0.05) but was not associated with fear (*r* = 0.038, *p* > 0.05). A significantly positive association between perception of dangerousness and fear was observed (*r* = 0.731, *p* < 0.01).

### 3.4. Path Analysis

**Figure 1:**
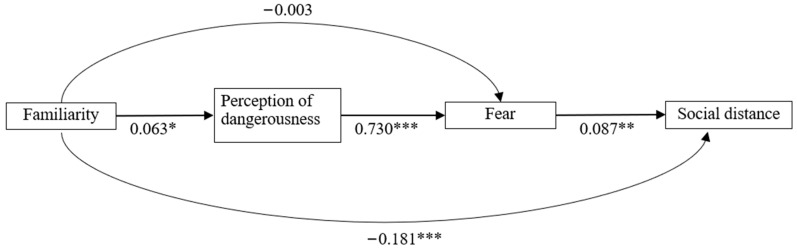
Hypothesized model relating familiarity, perception of dangerousness, fear, and social distance. * *p* < 0.05 ** *p* < 0.01 *** *p* < 0.001.

The hypothesized model relating familiarity, perception of dangerousness, fear, and social distance was tested using path analysis (Figure 1). The result showed a great fit (RMSEA = 0.040, CFI = 0.989, TLI = 0.966). Familiarity was negatively associated with social distance (*p* < 0.001) and positively associated with perception of dangerousness (*p* < 0.05). Perception of dangerousness was positively associated with fear (*p* < 0.001). Fear was positively associated with social distance (*p* < 0.01). As shown in Table 4, the indirect effect of perception of dangerousness on social distance through fear was reported to be significant (standardized indirect effect = 0.063, *p* < 0.01), but the indirect effect of familiarity on social distance was reported to be insignificant (standardized indirect effect = 0.004, *p* > 0.05).

In brief, the significant associations observed among familiarity, perception of dangerousness, fear, and social distance support the first three hypotheses. Nevertheless, the results do not fully support the last hypothesis that perception of dangerousness and fear mediate the association between familiarity and social distance.

## 4. Discussion

To the best of our knowledge, this study represents one of the first attempts to investigate Corrigan’s attributional model of mental-health-related stigma in Chinese society. This study adds to the rapidly expanding field of mental-health-related stigma, conceptualized as prejudicial attitudes, negative emotional reactions, and discriminatory behaviors in East Asia, where Confucian ideology is highly valued and mental illness is considered as a family misfortune, genetic taint, or past misdeeds [29,43,44,45]. The results of the study reveal that Chinese college students have moderate to high levels of stigma towards PMI, which concurs with previous studies [46,47,48].

A noteworthy observation lies in the low familiarity with PMI among participants, although evidence has shown that 20% to 30% of Chinese college students have experienced depressive symptoms [49]. One possible explanation is that Chinese people are inclined to hide their mental health problems in an effort to avoid ‘losing face’, associated with feelings of shame and a need to maintain a respectable standing and social image [29,45,50]. College students with mental illness, especially those without a formal diagnosis, are often exposed to the dilemma of disclosing their situation [51]. On the one hand, they may wish to seek support or receive help from others, such as peers, romantic partners, and instructors. On the other hand, they may be concerned with the risk of stigmatization and social exclusion after disclosing their problems because of the public misconceptions about mental illness [52,53]. Furthermore, it was reported that the utilization of mental health services and the intention to seek professional help for mental health problems were not high globally [54,55,56].

Images of violent acts committed by PMI portrayed by media increase belief in dangerousness and fear reactions [57]. It was found that college students expressed negative attitudes towards PMI because of media reports about violent behaviors committed by PMI, even if they did not observe the behaviors directly [24]. To be specific, a stronger desire for social distance was observed among female compared students to male students, a finding which is at odds with findings in Western countries documenting that female adults were less prone to displaying stigmatizing attitudes and behaviors towards mental illness [7,58,59]. However, our finding coincides with other studies conducted in other Chinese populations [60,61,62]. The discrepancy based on gender might be due to cultural differences. As noted earlier, Chinese culture is strongly rooted in Confucian ideology, attaching importance to collectivism, family reputation, saving face, and solidarity [29,44,45]. Females have a lower status than males under the patriarchal ideology of Confucianism. Females are expected to adhere to the cultural norms without expressing complaints, thereby resulting in an elevated level of negative attitudes towards PMI among females [61]. Contrary to prior research on the negative association between educational level and stigma in the general public [61,63], postgraduate students in this study reported less familiarity, more social distance, and more fear towards PMI than undergraduate students. It is possible that postgraduate students, who tend to focus more on their study and less on participating in extracurricular activities, including mental health education, are prone to developing a negative view about PMI [64]. Evidence has shown that low levels of mental health literacy are associated with more mental health stigma [65].

In line with the finding by Corrigan and his team [12], participants with more familiarity displayed less social distance towards PMI. Several studies revealed that individuals who had contact with PMI, such as family members and friends, were more likely to develop a better understanding of mental illness [66,67]. For instance, family members of PMI were more tolerant of their bizarre or combative behaviors and had less social distance [68]. Surprisingly, our finding suggests that familiarity is positively correlated with perception of dangerousness, which is not consistent with the first hypothesis. Corrigan and Nieweglowski’s [69] research review showed that despite a large body of evidence supporting an inverse correlation between familiarity and mental health stigma, a small number of studies found that more familiarity was associated with higher levels of stigma. Interestingly, most of the studies reporting positive relationships between familiarity and stigma were conducted among adolescents or college students [17,70,71]. A possible explanation is that adolescents and young adults, including college students, do not have sufficient mental health literacy to enable them to develop an empathic understanding for PMI with whom they are familiar.

Similar to other countries, the bivariate correlation and path analysis supported the assumption of the attributional model of stigma in China, namely that perception of dangerousness is positively linked to negative emotion reaction and consequently associated with behavioral discrimination [13]. It was validated that fear might work as an automatic response to perceived dangerousness [4]. Furthermore, driven by the perception that PMI are dangerous and violent, individuals might believe that PMI should be segregated from the community, thereby leading to social avoidance or social distance, a significant type of discriminatory behavioral response [13].

The hypothesis that perception of dangerousness and fear mediate the association between familiarity and social distance was not fully supported in our findings. This may partly be attributable to the more important role of contact quality instead of familiarity or contact level in stigma reduction [36,57,72,73]. According to the contact hypothesis, prejudice towards an ‘out-group’ could be alleviated effectively through four positive conditions, including equal status, common goals, and intergroup and authority support, indicating the vital role of contact quality in stigma reduction [69,72,74]. A prior study suggested that enhancing contact quality, with an emphasis on equal, voluntary, intimate, cooperative, pleasant, and positive contact, contributes to lower perceived dangerousness and fear among mental health professionals [41]. Conversely, familiarity was not necessarily associated with less negative attitudes towards PMI; for example, Fang et al. [36] found that family caregivers who had more familiarity might also endorse severe prejudicial attitudes and discriminatory behaviors towards PMI. Therefore, the development and implementation of a contact-based program should focus not only on promoting familiarity but also enhancing contact in multiple aspects (e.g., contact quality) [36].

There are a few limitations that should be acknowledged. First, given that this study employed a cross-section design, the causal mechanism among the main factors remains to be explored. As such, a longitudinal study that establishes the temporal ordering of key variables is essential to offer greater clarity on the interrelationships among the variables. The second limitation is the use of single-item measures of perception of dangerousness and fear; thus, further work is needed using more reliable methods for assessing these factors. Third, the survey was conducted in Shanghai, a major metropolitan area in China where the per capita income is the highest in the country. Therefore, our sample cannot represent the entire population of Chinese college students. Also, the online survey was conducted for two months only, resulting in a limited sample size. To increase the sample size, extending the timeline of the investigation from various cities in China should be considered in the future.

Despite the above limitations, this study has some implications for the development of interventions. In consideration of the vital role of traditional Chinese beliefs in stigmatization, greater efforts are required to design and develop culture-specific anti-stigma interventions [29]. For instance, “restoring face” was proposed as a unique strategy to challenge stigma in Chinese societies [50]. Specifically, the positive association found between familiarity and perception of dangerousness and the lack of mediating effects of perception of dangerousness and fear suggested that familiarity with PMI in itself is not sufficient to address negative emotional responses to PMI in order to reduce social distance. Future work should not only explore multiple elements of contacts (e.g., quality of contact) between PMI and their non-PMI peers but also consider local cultural factors (e.g., collectivism) to design interventions that address the stigma of mental illness [36].

## Figures and Tables

**Table 1:** Sociodemographic characteristics.

Characteristics	N/Mean	%/SD
Age	21.53	2.3
Gender		
Male	664	44.5
Female	829	55.5
Educational level		
≤Undergraduate	1026	68.7
Postgraduate	467	31.3
Hometown		
Urban area	980	65.6
Rural area	382	25.6
Others	131	8.8
Ethnicity		
Han	1442	96.6
Minority	51	3.4
Familiarity (mean ± SD score)	5.83 ± 3.32	
Perception of dangerousness (mean ± SD score)	5.24 ± 1.97	
Fear (mean ± SD score)	4.98 ± 2.15	
Social distance (mean ± SD score)	22.51 ± 5.96	

**Table 2:** Differences in main variables among students.

	Familiarity				Perception of Dangerousness				Fear				Social Distance			
	Mean	SD	*p*		Mean	SD	*p*		Mean	SD	*p*		Mean	SD	*p*	
Age			0.999				0.009	**			0.008	**			0.816	
Gender			0.008	**			0.069				0.991				<0.001	***
Male	6.08	3.43			5.34	2.04			4.98	2.23			21.33	6.38		
Female	5.62	3.21			5.16	1.91			4.98	2.08			23.45	5.428		
Educational level			<0.001	***			0.089				0.009	**			<0.001	***
≤Undergraduate	6.09	3.35			5.18	2.02			4.88	2.16			22.03	6.151		
Postgraduate	5.24	3.17			5.37	1.86			5.19	2.10			23.55	5.39		
Hometown			0.36				0.994				0.437				0.899	
Urban area	5.91	3.29			5.24	2.00			4.93	2.17			22.53	6.22		
Rural area	5.63	3.35			5.25	1.94			5.08	2.08			22.39	5.32		
Others	5.79	3.43			5.25	1.82			5.07	2.15			22.63	5.81		
Ethnicity			0.995				0.086				0.84				0.428	
Han	5.83	3.32			5.22	1.97			4.98	2.14			22.48	6.00		
Minority	5.82	3.21			5.71	1.97			4.92	2.37			23.16	4.781		

** *p* < 0.01 *** *p* < 0.001.

**Table 3:** Bivariate correlations among main variables.

	1	2	3	4
1. Familiarity	1			
2. Perception of dangerousness	0.063 *	1		
3. Fear	0.039	0.731 **	1	
4. Social distance	−0.194 **	0.065 *	0.083 **	1

* *p* < 0.05 ** *p* < 0.01.

**Table 4:** Direct and indirect effects of familiarity on social distance.

		95% Confidence Intervals	
Pathways	Standardized Indirect Effect	Lower Limit	Upper Limit
PD → F2 → SD	0.063 **	0.029	0.101
F1 → PD → F2 → SD	0.004	0.001	0.008

F1 = familiarity; PD = perception of dangerousness; F2 = fear; SD = social distance; ** *p* < 0.01.

## Data Availability

All data that support the findings of this study are available on request from the corresponding author. The data are not publicly available due to privacy or ethical restrictions.

## References

[1] Costa A.C.D.S., Menon V., Phadke R., Dapke K., Miranda A.V., Ahmad S., Essar M.Y., Hashim H.T. (2022). Mental health in the post COVID-19 era: Future perspectives. Einstein São Paulo.

[2] Vadivel R., Shoib S., El Halabi S., El Hayek S., Essam L., Gashi Bytyçi D., Karaliuniene R., Schuh Teixeira A.L., Nagendrappa S., Ramalho R. (2021). Mental health in the post-COVID-19 era: Challenges and the way forward. Gen. Psychiatry.

[3] Withers M., Jahangir T., Kubasova K., Ran M.-S. (2022). Reducing stigma associated with mental health problems among university students in the Asia-Pacific: A video content analysis of student-driven proposals. Int. J. Soc. Psychiatry.

[4] Corrigan P.W., Rowan D., Green A., Lundin R., River P., Uphoff-Wasowski K., White K., Kubiak M.A. (2002). Challenging Two Mental Illness Stigmas: Personal Responsibility and Dangerousness. Schizophr. Bull..

[5] Livingston J.D., Boyd J.E. (2010). Correlates and consequences of internalized stigma for people living with mental illness: A systematic review and meta-analysis. Soc. Sci. Med..

[6] Goffman E. (1963). Stigma: Notes on the Management of Spoiled Identity.

[7] Clement S., Schauman O., Graham T., Maggioni F., Evans-Lacko S., Bezborodovs N., Morgan C., Rüsch N., Brown J.S.L., Thornicroft G. (2015). What is the impact of mental health-related stigma on help-seeking? A systematic review of quantitative and qualitative studies. Psychol. Med..

[8] Corrigan P.W. (2012). Where Is the Evidence Supporting Public Service Announcements to Eliminate Mental Illness Stigma?. Psychiatr. Serv..

[9] Henderson C., Evans-Lacko S., Thornicroft G. (2013). Mental Illness Stigma, Help Seeking, and Public Health Programs. Am. J. Public Health.

[10] Xu Z., Müller M., Heekeren K., Theodoridou A., Metzler S., Dvorsky D., Oexle N., Walitza S., Rössler W., Rüsch N. (2016). Pathways between stigma and suicidal ideation among people at risk of psychosis. Schizophr. Res..

[11] Weiner B. (1995). Judgments of Responsibility: A Foundation for a Theory of Social Conduct.

[12] Corrigan P.W., Green A., Lundin R., Kubiak M.A., Penn D.L. (2001). Familiarity with and Social Distance from People Who Have Serious Mental Illness. Psychiatr. Serv..

[13] Corrigan P., Markowitz F.E., Watson A., Rowan D., Kubiak M.A. (2003). An Attribution Model of Public Discrimination Towards Persons with Mental Illness. J. Health Soc. Behav..

[14] Angermeyer M.C., Matschinger H., Corrigan P.W. (2004). Familiarity with mental illness and social distance from people with schizophrenia and major depression: Testing a model using data from a representative population survey. Schizophr. Res..

[15] Alexander L., Link B. (2003). The impact of contact on stigmatizing attitudes toward people with mental illness. J. Ment. Health.

[16] Link B.G., Cullen F.T. (1986). Contact with the Mentally Ill and Perceptions of How Dangerous They Are. J. Health Soc. Behav..

[17] Corrigan P.W., Lurie B.D., Goldman H.H., Slopen N., Medasani K., Phelan S. (2005). How Adolescents Perceive the Stigma of Mental Illness and Alcohol Abuse. Psychiatr. Serv..

[18] Janulis P., Ferrari J.R., Fowler P. (2013). Understanding public stigma toward substance dependence: Public stigma toward dependence. J. Appl. Soc. Psychol..

[19] Werner P., Kalaitzaki A.E., Spitzer N., Raviv-Turgeman L., Koukouli S., Tziraki C. (2019). Stigmatic beliefs towards persons with dementia: Comparing Israeli and Greek college students. Int. Psychogeriatr..

[20] Liu X., Pan L., Cao J. (2017). Effectiveness of a group intervention with aims of reducing stigma towards mental illness in nursing students. J. Nurses Train..

[21] Crisp A., Gelder M., Goddard E., Meltzer H. (2005). Stigmatization of people with mental illnesses: A follow-up study within the Changing Minds campaign of the Royal College of Psychiatrists. World Psychiatry.

[22] Yamaguchi S., Wu S.-I., Biswas M., Yate M., Aoki Y., Barley E.A., Thornicroft G. (2013). Effects of Short-Term Interventions to Reduce Mental Health–Related Stigma in University or College Students: A Systematic Review. J. Nerv. Ment. Dis..

[23] Babicki M., Małecka M., Kowalski K., Bogudzińska B., Piotrowski P. (2021). Stigma Levels Toward Psychiatric Patients Among Medical Students—A Worldwide Online Survey Across 65 Countries. Front. Psychiatry.

[24] Luo A., He H., Mohamed S., Rosenheck R. (2018). Medical Student Attitudes Towards People with Mental Illness in China: A Qualitative Study. Cult. Med. Psychiatry.

[25] Lee A.A., Laurent S.M., Wykes T.L., Kitchen Andren K.A., Bourassa K.A., McKibbin C.L. (2014). Genetic attributions and mental illness diagnosis: Effects on perceptions of danger, social distance, and real helping decisions. Soc. Psychiatry Psychiatr. Epidemiol..

[26] Lyndon A.E., Crowe A., Wuensch K.L., McCammon S.L., Davis K.B. (2019). College students’ stigmatization of people with mental illness: Familiarity, implicit person theory, and attribution. J. Ment. Health.

[27] Henderson N.L., Dressler W.W. (2017). Medical Disease or Moral Defect? Stigma Attribution and Cultural Models of Addiction Causality in a University Population. Cult. Med. Psychiatry.

[28] Krendl A.C., Pescosolido B.A. (2020). Countries and Cultural Differences in the Stigma of Mental Illness: The East–West Divide. J. Cross-Cult. Psychol..

[29] Ran M.-S., Hall B.J., Su T.T., Prawira B., Breth-Petersen M., Li X.-H., Zhang T.-M. (2021). Stigma of mental illness and cultural factors in Pacific Rim region: A systematic review. BMC Psychiatry.

[30] Tsang H.W.H., Angell B., Corrigan P.W., Lee Y.-T., Shi K., Lam C.S., Jin S., Fung K.M.T. (2007). A cross-cultural study of employers’ concerns about hiring people with psychotic disorder: Implications for recovery. Soc. Psychiatry Psychiatr. Epidemiol..

[31] Heim E., Henderson C., Kohrt B.A., Koschorke M., Milenova M., Thornicroft G. (2020). Reducing mental health-related stigma among medical and nursing students in low- and middle-income countries: A systematic review. Epidemiol. Psychiatr. Sci..

[32] Wada M., Suto M.J., Lee M., Sanders D., Sun C., Le T.N., Goldman-Hasbun J., Chauhan S. (2019). University students’ perspectives on mental illness stigma. Ment. Health Prev..

[33] Holmes E.P., Corrigan P.W., Williams P., Canar J., Kubiak M.A. (1999). Changing Attitudes About Schizophrenia. Schizophr. Bull..

[34] Link B.G., Cullen F.T., Frank J., Wozniak J.F. (1987). The Social Rejection of Former Mental Patients: Understanding Why Labels Matter. Am. J. Sociol..

[35] James B.O., Omoaregba J.O., Okogbenin E.O. (2012). Stigmatising attitudes towards persons with mental illness: A survey of medical students and interns from Southern Nigeria. Ment. Illn..

[36] Fang Q., Zhang T.-M., Wong Y.L.I., Yau Y.Y., Li X.-H., Li J., Chui C.H.K., Tse S., Chan C.L.-W., Chen E.Y.H. (2021). The mediating role of knowledge on the contact and stigma of mental illness in Hong Kong. Int. J. Soc. Psychiatry.

[37] Corrigan P.W., Powell K.J., Michaels P.J. (2014). Brief battery for measurement of stigmatizing versus affirming attitudes about mental illness. Psychiatry Res..

[38] Li X.-H., Zhang T.-M., Yau Y.Y., Wang Y.-Z., Wong Y.-L.I., Yang L., Tian X., Chan C.L.-W., Ran M.-S. (2021). Peer-to-peer contact, social support and self-stigma among people with severe mental illness in Hong Kong. Int. J. Soc. Psychiatry.

[39] Lauber C., Nordt C., Falcato L., Rössler W. (2004). Factors Influencing Social Distance Toward People with Mental Illness. Community Ment. Health J..

[40] Hooper D., Couglan J., Mullen M.R. (2008). Structural equation modelling: Guidelines for determining model fit. Electron. J. Bus. Res. Methods.

[41] Ran M.-S., Peng M.-M., Yau Y.Y., Zhang T.-M., Li X.-H., Wong I.Y.L., Ng S., Thornicroft G., Chan C.L.-W., Lu L. (2022). Knowledge, contact and stigma of mental illness: Comparing three stakeholder groups in Hong Kong. Int. J. Soc. Psychiatry.

[42] Werner P., Raviv-Turgeman L., Corrigan P.W. (2020). The influence of the age of dementia onset on college students’ stigmatic attributions towards a person with dementia. BMC Geriatr..

[43] Abdullah T., Brown T.L. (2011). Mental illness stigma and ethnocultural beliefs, values, and norms: An integrative review. Clin. Psychol. Rev..

[44] Haraguchi K., Maeda M., Mei Y.X., Uchimura N. (2009). Stigma associated with schizophrenia: Cultural comparison of social distance in Japan and China: Stigma associated with schizophrenia. Psychiatry Clin. Neurosci..

[45] Yang L.H. (2007). Application of mental illness stigma theory to chinese societies: Synthesis and new directions. Singap. Med. J..

[46] Kong L., Fan W., Xu N., Meng X., Qu H., Yu G. (2020). Stigma Among Chinese Medical Students Toward Individuals With Mental Illness. J. Psychosoc. Nurs. Ment. Health Serv..

[47] Mak W., Cheung F., Wong S., Tang W., Lau J., Woo J., Lee D. (2015). Stigma towards people with psychiatric disorders. Hong Kong Med. J..

[48] Yin H., Wardenaar K.J., Xu G., Tian H., Schoevers R.A. (2020). Mental health stigma and mental health knowledge in Chinese population: A cross-sectional study. BMC Psychiatry.

[49] Gao L., Xie Y., Jia C., Wang W. (2020). Prevalence of depression among Chinese university students: A systematic review and meta-analysis. Sci. Rep..

[50] Yang L.H., Kleinman A. (2008). ‘Face’ and the embodiment of stigma in China: The cases of schizophrenia and AIDS. Soc. Sci. Med..

[51] Taniguchi E. (2022). The roles of mental illness disclosure and disclosure strategies on well-being among college students. J. Am. Coll. Health.

[52] Corrigan P.W., Rao D. (2012). On the Self-Stigma of Mental Illness: Stages, Disclosure, and Strategies for Change. Can. J. Psychiatry.

[53] Woodhead E.L., Chin-Newman C., Spink K., Hoang M., Smith S.A. (2021). College students’ disclosure of mental health problems on campus. J. Am. Coll. Health.

[54] Ning X., Wong J.P.-H., Huang S., Fu Y., Gong X., Zhang L., Hilario C., Fung K.P.-L., Yu M., Poon M.K.-L. (2022). Chinese University Students’ Perspectives on Help-Seeking and Mental Health Counseling. Int. J. Environ. Res. Public Health.

[55] Osborn T.G., Li S., Saunders R., Fonagy P. (2022). University students’ use of mental health services: A systematic review and meta-analysis. Int. J. Ment. Health Syst..

[56] Pan Q., Hao Z. (2023). Chinese college students’ help-seeking behavior: An application of the modified theory of planned behavior. PsyCh J..

[57] Jorm A.F., Reavley N.J., Ross A.M. (2012). Belief in the dangerousness of people with mental disorders: A review. Aust. N. Z. J. Psychiatry.

[58] Mackenzie C.S., Visperas A., Ogrodniczuk J.S., Oliffe J.L., Nurmi M.A. (2019). Age and sex differences in self-stigma and public stigma concerning depression and suicide in men. Stigma Health.

[59] Sirey J.A., Bruce M.L., Alexopoulos G.S., Perlick D.A., Raue P., Friedman S.J., Meyers B.S. (2001). Perceived Stigma as a Predictor of Treatment Discontinuation in Young and Older Outpatients With Depression. Am. J. Psychiatry.

[60] Chan S.K.W., Tam W.W.Y., Lee K.W., Hui C.L.M., Chang W.C., Lee E.H.M., Chen E.Y.H. (2016). A population study of public stigma about psychosis and its contributing factors among Chinese population in Hong Kong. Int. J. Soc. Psychiatry.

[61] Lo L.L.H., Suen Y.N., Chan S.K.W., Sum M.Y., Charlton C., Hui C.L.M., Lee E.H.M., Chang W.C., Chen E.Y.H. (2021). Sociodemographic correlates of public stigma about mental illness: A population study on Hong Kong’s Chinese population. BMC Psychiatry.

[62] Suen Y.N., Chan K.W.S., Siu L.T.T., Lo L.H.L., Cheung C., Hui L.M.C., Lee H.M.E., Chang W.C., Wong P.S., Chen Y.H.E. (2021). Relationship between stressful life events, stigma and life satisfaction with the willingness of disclosure of psychotic illness: A community study in Hong Kong. Early Interv. Psychiatry.

[63] Girma E., Tesfaye M., Froeschl G., Möller-Leimkühler A.M., Müller N., Dehning S. (2013). Public Stigma against People with Mental Illness in the Gilgel Gibe Field Research Center (GGFRC) in Southwest Ethiopia. PLoS ONE.

[64] Wu R., He X.S. (2013). The Influence of College Students ’Mental Health Literacy on Professional Help-seeking Tendency: Take Shanghai E School for Example. J. East China Univ. Sci. Technol. Soc. Sci. Ed..

[65] Rafal G., Gatto A., DeBate R. (2018). Mental health literacy, stigma, and help-seeking behaviors among male college students. J. Am. Coll. Health.

[66] Steiger S., Sowislo J.F., Moeller J., Lieb R., Lang U.E., Huber C.G. (2022). Personality, self-esteem, familiarity, and mental health stigmatization: A cross-sectional vignette-based study. Sci. Rep..

[67] Subramaniam M., Abdin E., Picco L., Pang S., Shafie S., Vaingankar J.A., Kwok K.W., Verma K., Chong S.A. (2017). Stigma towards people with mental disorders and its components—A perspective from multi-ethnic Singapore. Epidemiol. Psychiatr. Sci..

[68] Wang M., Wang Y., Xu J., Meng N., Li X., Liu Z., Huang J. (2021). Individual-level socioeconomic status and contact or familiarity with people with mental illness: A cross-sectional study in Wuhou District, Chengdu, Southwest China. BMC Fam. Pract..

[69] Corrigan P.W., Nieweglowski K. (2019). How does familiarity impact the stigma of mental illness?. Clin. Psychol. Rev..

[70] Batastini A.B., Bolanos A.D., Morgan R.D. (2014). Attitudes toward hiring applicants with mental illness and criminal justice involvement: The impact of education and experience. Int. J. Law Psychiatry.

[71] Phelan J.E., Basow S.A. (2007). College Students’ Attitudes Toward Mental Illness: An Examination of the Stigma Process. J. Appl. Soc. Psychol..

[72] Allport G.W. (1954). The Nature of Prejudice.

[73] Pettigrew T.F., Tropp L.R., Wagner U., Christ O. (2011). Recent advances in intergroup contact theory. Int. J. Intercult. Relat..

[74] Ran M.-S., Wang Y.-Z., Lu P.-Y., Weng X., Zhang T.-M., Deng S.-Y., Li M., Luo W., Wong I.Y.-L., Yang L.H. (2022). Effectiveness of enhancing contact model on reducing stigma of mental illness among family caregivers of persons with schizophrenia in rural China: A cluster randomized controlled trial. Lancet Reg. Health West. Pac..

